# A report of use of baclofen in intractable hiccups

**DOI:** 10.1192/j.eurpsy.2021.2058

**Published:** 2021-08-13

**Authors:** R. Dhakad, V. Niranjan, P. Rastogi, V. Pal

**Affiliations:** 1 Psychiatry, MGM Medical College, Indore, India; 2 Psychiatry, MGM Medical College, INDORE, India

**Keywords:** baclofen, intractable hiccups, schizophrénia, GABA adrenergic

## Abstract

**Introduction:**

Hiccups are an involuntarily powerful spasm of the diaphragm, followed by a sudden inspiration with a closure of the glottis. Hiccups lasting longer than one month is termed intractable hiccups. Intractable hiccups can be caused by structural or functional disturbances of the medulla, afferent or efferent nerves to the respiratory muscles or metabolic and endocrine disorders, drugs, general anaesthesia and emotional problems.

**Objectives:**

Authors present a case report about curing a patient of intractable hiccups using baclofen along with literature review.

**Methods:**

A case report along with literature review forms the basis of discussion.

**Results:**

A 30-year female diagnosed with schizophrenia stable on 2mg risperidone for 3 years presented to the outpatient department with complain of intractable hiccups for 6 months. Frequency of hiccups was around 10-12 times per minute and continued throughout the day leading to significant socio-occupational distress. patient had been receiving medical treatment for last 4 months for the same including Metoclopramide, chlorpromazine along with trying breath holding and drinking cold water but symptoms persisted. Her ECG, chest X-ray, complete blood counts were unremarkable, CT scan of brain was normal. Patient was started on baclofen 10mg thrice daily. Within 1-week patient had dramatic response and complete remission was achieved in 2 weeks.

**Conclusions:**

Beclofen is effective in hiccups because it is an analogue of GABA, that decreases excitability and inhibits the hiccup reflex, which reduces synaptic transmission. Baclofen is used to treat hiccups, and can be used either as a first-line treatment or if patient does not respond to other medications.

**Disclosure:**

No significant relationships.

